# Biocompatible Palladium Nanoparticles Prepared Using Vancomycin for Colorimetric Detection of Hydroquinone

**DOI:** 10.3390/polym15143148

**Published:** 2023-07-24

**Authors:** Shoubei Gao, Kai Liu, Xianbing Ji, Yanshuai Cui, Ruyu Li, Guanglong Ma, Yongqiang Zhang, Longgang Wang

**Affiliations:** 1 State Key Laboratory of Metastable Materials Science and Technology, Hebei Key Laboratory of Nano-Biotechnology, Hebei Key Laboratory of Applied Chemistry, Yanshan University, Qinhuangdao 066004, China; 18903327331@163.com (S.G.); kailiu241819@163.com (K.L.); liruyuys@163.com (R.L.); 2 Department of Environmental Engineering, Hebei University of Environmental Engineering, Qinhuangdao 066102, China; jixianbing@hebuee.edu.cn (X.J.); cuiyanshuai09@163.com (Y.C.); 3 Centre for Cancer Immunology, Faculty of Medicine, University of Southampton, Southampton SO16 6YD, UK; mguanglong@gmail.com

**Keywords:** nanozyme, vancomycin, palladium, nanoparticles, detection

## Abstract

Hydroquinone poses a major threat to human health and is refractory to degradation, so it is important to establish a convenient detection method. In this paper, we present a novel colorimetric method for the detection of hydroquinone based on a peroxidase-like Pd nanozyme. The vancomycin-stabilized palladium nanoparticles (Van-Pd_n_ NPs, n = 0.5, 1, 2) were prepared using vancomycin as a biological template. The successful synthesis of Van-Pd_n_ NPs (n = 0.5, 1, 2) was demonstrated by UV-vis spectrophotometry, transmission electron microscopy, and X-ray diffraction. The sizes of Pd nanoparticles inside Van-Pd_0.5_ NPs, Van-Pd_1_ NPs, and Van-Pd_2_ NPs were 2.6 ± 0.5 nm, 2.9 ± 0.6 nm, and 4.3 ± 0.5 nm, respectively. Furthermore, Van-Pd_2_ NPs exhibited excellent biocompatibility based on the MTT assay. More importantly, Van-Pd_2_ NPs had good peroxidase-like activity. A reliable hydroquinone detection method was established based on the peroxidase-like activity of Van-Pd_2_ NPs, and the detection limit was as low as 0.323 μM. Therefore, vancomycin improved the peroxidase-like activity and biocompatibility of Van-Pd_2_ NPs. Van-Pd_2_ NPs have good application prospects in the colorimetric detection of hydroquinone.

## 1. Introduction

Hydroquinone (HQ) is easy to react with peroxide free radicals and is commonly used in developers, hair dyes, pharmaceutical raw materials, and oxygen scavengers. However, HQ can inhibit the central nervous system and damage liver and kidney function, and it has been included in the list of three types of carcinogens. Therefore, it is necessary to establish an efficient and reliable HQ detection platform.

At present, there are many detection and analysis methods, such as colorimetric analysis [1,2], electrochemical analysis [3], and high-performance liquid chromatography [4]. Among them, the colorimetric analysis method has the advantages of easy operation, low cost, and good visibility. Therefore, colorimetry has become one of the main ways. In most colorimetric analyses of HQ, the addition of natural enzymes is required to catalyze the reaction. However, natural enzymes have disadvantages such as high cost and easy inactivation. Since the discovery of Fe_3_O_4_ nanozymes [5,6], a variety of nanomaterials have been found to have enzyme-like properties. Nanozymes have good stability, high efficiency, and easy acquisition, so they are widely used in colorimetric detection. Many nanomaterials, such as MnO_2_ [7], UiO-67-Cu^2+^ [8], Co_3_O_4_ nanoplates [9], and PPy NPs [10], have shown enzyme-like catalytic ability and have been used in colorimetric detection. Ge et al. [11] prepared MnO/PC nanohybrid material, which was used for colorimetric detection of HQ. The experiment yielded a desirable linear relationship within the range of 0–50 µM, and a detection limit of 0.5 µM was achieved. Because of the low activity of the artificial enzyme used in their study, the detection limit is high, and the detection range of the method is not wide enough. These limitations may impact the applicability and effectiveness of the method in certain contexts or when dealing with a wide range of concentrations.

Among the many nanomaterials developed by people, noble metal nanozymes are a class of nanomaterials that are widely studied [12,13,14]. The Pd@Pt nanoparticles prepared by Wang et al. [15] have three enzyme mimicry activities, and the catalytic activity can be regulated by DNA. Tang et al. [16] proposed the application of ultrathin Pd nanosheets as photo controllable peroxidase mimics. As a kind of precious metal, palladium nanozymes can also be prepared to explore their properties. Palladium nanoparticles [17] have the characteristics of a small size, a large specific surface area, and many active sites. The good catalytic activity of palladium nanoparticles is not only related to the advantages of the nanoparticles themselves but also to the stability and dispersion degree of the nanoparticles in the solution. In the process of preparing palladium nanozyme, it is necessary to prevent it from coagulating. The catalytic performance of palladium nanoparticles will be greatly reduced after coagulation.

Natural substances as biological templates can be used to greatly improve the biocompatibility of nanozymes [18]. The biological template method can provide us with new synthetic avenues to develop new functional materials with the advantages of complexity, hierarchy, and adaptability. The technology is widely used in metal oxides [19], ceramics [20], inorganic materials [21], organic semiconductors [22], and precious metal nanoparticles [23]. Vancomycin is a glycopeptide antibiotic with a molecular weight of 1486. Vancomycin has a molecular formula with a heptapeptide core and excellent antibacterial properties against gram-positive bacteria [24]. Vancomycin contains 9 hydroxyl groups, 2 amino groups, and 1 carboxyl group, which results in vancomycin being water-soluble. Vancomycin has the potential to be used as a template and stabilizer to efficiently load precious metal nanoparticles.

In this work, we prepared vancomycin-stabilized palladium nanoparticles (Van-Pd_n_ NPs, n = 0.5, 1, 2) using vancomycin as the biological template. The successful synthesis of Van-Pd_n_ NPs (n = 0.5, 1, 2) was characterized by UV-vis spectrophotometry, transmission electron microscopy, and X-ray diffraction. The enzyme-like activity and catalytic kinetics of the Van-Pd_2_ NPs were also studied. By utilizing the peroxidase-like activity of Van-Pd_2_ NPs, a simple and reliable detection platform for HQ was constructed. The measurement of HQ concentration in the drug using Van-Pd_2_ NPs in real samples confirms the great potential for biomedically relevant tests.

## 2. Materials and Methods

### 2.1. Materials

Vancomycin hydrochloride, sodium borohydride (NaBH_4_), hydroquinone (HQ), hydrogen peroxide (H_2_O_2_), 3,3′,5,5′-tetramethylbenzidine (TMB), dimethyl sulfoxide (DMSO), and thiazole blue (MTT) were purchased from Aladdin (Shanghai, China). Sodium tetrachloropalladate (Na_2_PdCl_4_) was purchased from West Asia Reagent (Chengdu, China).

### 2.2. Synthesis of Van-Pd_n_ NPs

An amount of 73 μL vancomycin hydrochloride (Van·HCl, 10 mM) solution and different amounts of Na_2_PdCl_4_ (10 mM) solution were taken into a 2 mL PE tube; the substance ratio of vancomycin to Na_2_PdCl_4_ was 2:1, 1:1, and 1:2, respectively. Then 1000 μL of deionized water was added. They were placed in a constant-temperature mixer at 25 °C and incubated for 12 h at 600 rpm. After 10 μL of NaBH_4_ solution (1 M, dissolved in 0.3 M NaOH solution) was added, they were placed in a constant temperature mixer for 12 h, and the Van-Pd_n_ NPs (n = 0.5, 1, 2) were obtained after dialysis.

### 2.3. Enzyme-like Activity Characterization of Van-Pd_n_ NPs

To determine peroxidase-like activity, 200 μL of Van-Pd_n_ NPs (C_Pd_ = 0.9 mM) and 300 μL of a 0.2 M pH = 4 acetic acid-sodium acetate buffer solution were mixed and incubated in a 2 mL PE tube. 1000 μL of a 0.2 M acetic acid-sodium acetate buffer solution containing 0.6 mM TMB and 100 μL of 0.03 M H_2_O_2_ were mixed and incubated in a constant temperature mixer at 25 °C at 600 rpm for 2 min. Finally, UV-vis spectrophotometry was used to determine absorbance.

To explore the optimal reaction temperature of Van-Pd_n_ NPs, 200 μL of Van-Pd_n_ NPs (C_Pd_ = 0.9 mM) and 300 μL of acetic acid-sodium acetate buffer solution at different pHs were added to a 2 mL PE tube, and 1000 μL of 0.2 M acetic acid-sodium acetate buffer solution at different pHs containing 0.6 mM TMB were mixed. The samples were incubated in a constant-temperature mixer at 25 °C at 600 rpm for 5 min. Finally, UV-vis spectrophotometry was used to determine the absorbance of the sample at 652 nm. The pH range was 1–12. The temperature range was 5–65 °C.

The catalytic kinetics of the system were studied using the following experimental approach or methodology: 200 μL of Van-Pd_n_ NPs (C_Pd_ = 0.9 mM) were added to a 2 mL PE tube. The absorbance of the sample over time at 652 nm was then tested using a UV-vis spectrophotometer. The amount of buffer solution was 1200–300 μL at 100 μL intervals; The amount of buffer solution containing TMB was 100–1000 μL at 100 μL intervals. The total amount of liquid was 1500 μL. The formula 1 was used to study the affinity for substrate and the maximum rate of the catalytic reaction during the catalytic process:

v = *V_max_*[S]/(*K_m_* + [S]) (1)

Symbol description: *K_m_*—Michael’s constant; *V_max_*—maximum reaction rate; [S]—substrate concentration.

Furthermore, the peroxidase-like mechanism was tested using terephthalic acid (TA) as a fluorescent probe with the following steps. First, four experimental groups were established: TA, TA + Van-Pd_n_ NPs, TA + H_2_O_2_ and TA + H_2_O_2_ + Van-Pd_n_ NPs. As for TA + H_2_O_2_ + Van-Pd_n_ NPs, 1000 µL of TA (C_TA_ = 0.5 mM) was added into a 2 mL PE tube, and subsequently, 200 µL of an acetic acid-sodium acetate buffer solution with a pH of 4 was introduced into the same PE tube. The purpose of adding the buffer solution is to maintain a stable pH environment within the tube. This ensures that the conditions remain optimal for any subsequent steps or reactions that may take place. The fluorescence spectra of the final solution were measured using a fluorescence spectrometer to analyze the emission of light from the samples. The other control groups underwent the same experimental procedure as the samples being analyzed.

### 2.4. Determination of HQ Concentration

An amount of 50 μL of Van-Pd_n_ NPs (n = 0.5, 1, 2, C_Pd_ = 0.9 mM), 1000 μL of 0.2 M, pH = 3, acetic acid-sodium acetate buffer solution containing 0.6 mM TMB, 100 μL of 0.03 M solution of H_2_O_2_, and 200 μL of HQ solution of different concentrations were mixed in a 2 mL PE tube, and then the PE tube was placed in a constant temperature mixer for 5 min at 35 °C and 600 rpm. Finally, its absorbance was measured using a UV-vis spectrophotometer. The relationship between the difference in absorbance and concentration was used as the standard curve for detecting HQ. HQ solution at a concentration of 0–10 mM was added to PE tubes. HQ recovery in tap water and seawater was detected using the established standard curve.

An amount of 50 μL of Van-Pd_n_ NPs (n = 0.5, 1, 2, C_Pd_ = 0.9 mM), 1000 μL of 0.2 M, pH = 3, acetic acid-sodium acetate buffer solution containing 0.6 mM TMB, 100 μL of 0.03 M solution of H_2_O_2_, and 200 μL of HQ solution of different concentrations were added to a 2 mL PE tube. The 2 mL PE tube was placed in a constant-temperature mixer at 30 °C and 600 rpm for 5 min. Finally, the absorbance was measured using a UV-vis spectrophotometer. The recovery of the sample was calculated using the spike recovery formula [25].

The selectivity of Van-Pd_2_ NPs was investigated by detecting hydroquinone and potentially interfering substances, such as Mg^2+^, alanine (Ala), phenylalanine (Phe), leucine (Leu), glycine (Gly), proline (Pro), glutamic acid (Glu), maltose (Mal), lactose (Lac), and fructose (Fru). The experimental process was the same as above; the concentration of HQ is 1 mM, and the concentration of other interfering substances is 10 mM.

### 2.5. Biocompatibility of Van-Pd_n_ NPs

The biocompatibility of Van-Pd_n_ NPs (n = 0.5, 1, 2) was determined by MTT. First, cells were added to a 96-well plate at a concentration of 5.0 × 10^3^ per well and incubated for 24 h. The DMEM medium containing nanozymes was then replaced with the original medium and incubated for 24 h. The medium was replaced with a thiazole blue (MTT) solution and incubated for 4 h. Finally, the MTT solution was replaced with dimethyl sulfoxide (DMSO), and the absorbance of the 96-well plate was determined using a multifunctional microplate reader.

## 3. Results and Discussion

### 3.1. Characterization of Van-Pd_n_ NPs

**Figure 1:**
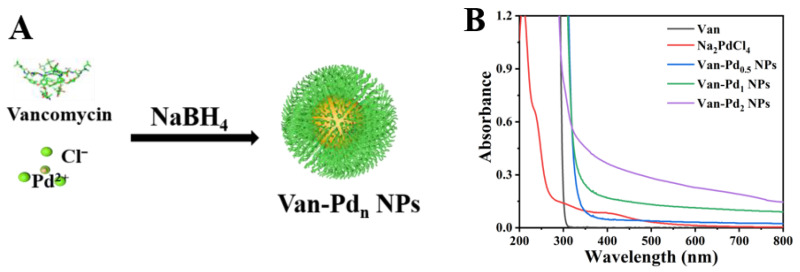
(**A**) The preparation method and (**B**) a graph with the absorption spectrum from the UV-Vis spectrophotometer of Van-Pd_n_ NPs (n = 0.5, 1, 2).

The synthesis of Van-Pd_n_ NPs was first characterized by UV-vis spectrophotometry. Figure 1A shows the preparation method of Van-Pd_n_ NPs. As shown in Figure 1B, Na_2_PdCl_4_ has an absorption peak at 420 nm in the UV-vis spectrum, which is caused by Pd^2+^. However, there is no characteristic absorption peak of Na_2_PdCl_4_ at 420 nm in the spectra of Van-Pd_0.5_ NPs, Van-Pd_1_ NPs, and Van-Pd_2_ NPs. When Pd^2+^ is reduced to Pd atoms, the absorption peak at 420 nm disappears [26]. Therefore, Pd^2+^ is reduced to Pd atoms in the process of synthesizing Van-Pd_n_ NPs. This indicates the successful preparation of Van-Pd_n_ NPs.

**Figure 2:**
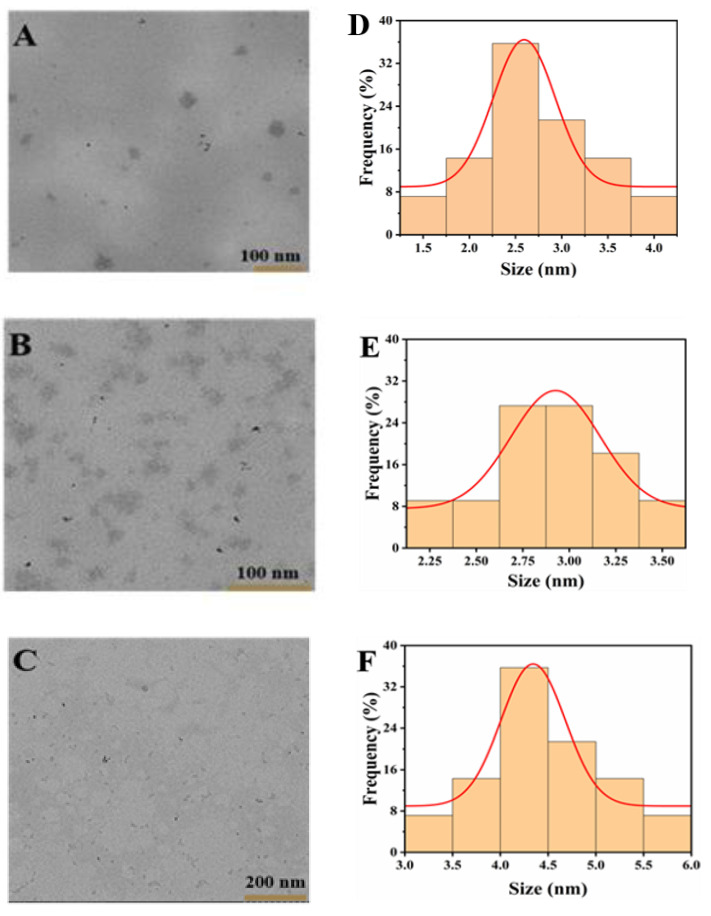
TEM image and statistic size: (**A**,**D**) Van-Pd_0.5_ NPs, (**B**,**E**) Van-Pd_1_ NPs, and (**C**,**F**) Van-Pd_2_, respectively.

Figure 2 shows TEM images of Van-Pd_n_ NPs (n = 0.5, 1, 2). Van-Pd_0.5_ NPs, Van-Pd_1_ NPs, and Van-Pd_2_ NPs have good dispersion and a small particle size. Van-Pd_0.5_ NPs have a particle size of 2.6 ± 0.5 nm, Van-Pd_1_ NPs have a particle size of 2.9 ± 0.6 nm, and Van-Pd_2_ NPs have a particle size of 4.3 ± 0.5 nm.

**Figure 3:**
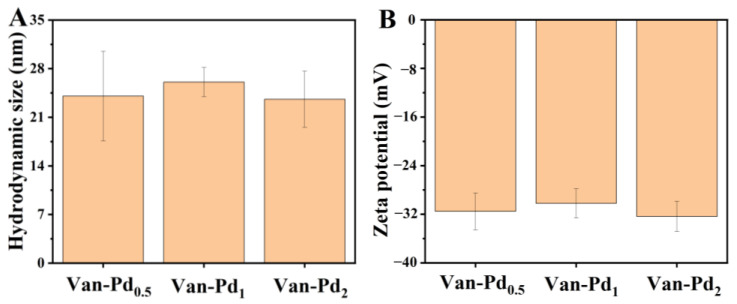
(**A**) DLS characterization of Van-Pd_n_ NPs (n = 0.5, 1, 2): (**A**) hydrodynamic size and (**B**) zeta potential.

DLS is a commonly used way to characterize the hydrodynamic size and zeta potential of nanoparticles in water [27]. Since most of the reaction processes, such as the catalysis of nanozymes, are carried out in aqueous solutions, DLS testing of Van-Pd_n_ NPs (n = 0.5, 1, 2) is required. As can be seen in Figure 3A, the hydrodynamic sizes of nanoparticles of Van-Pd_0.5_ NPs, Van-Pd_1_ NPs, and Van-Pd_2_ NPs were 24.1 nm, 26.1 nm, and 23.6 nm, respectively. The hydrodynamic size of Van-Pd_n_ NPs (n = 0.5, 1, 2) with different molar ratios did not have an obvious difference. Moreover, the zeta potential of the Van-Pd_n_ NPs was tested. As can be seen from Figure 3B, the zeta potentials of Van-Pd_0.5_ NPs, Van-Pd_1_ NPs, and Van-Pd_2_ NPs were −31.5 mV, −30.2 mV, and −32.4 mV, respectively. Among the Van-Pd_n_ NPs (n = 0.5, 1, 2), Van-Pd_2_ NPs had the largest absolute value of zeta potential in aqueous solution.

**Figure 4:**
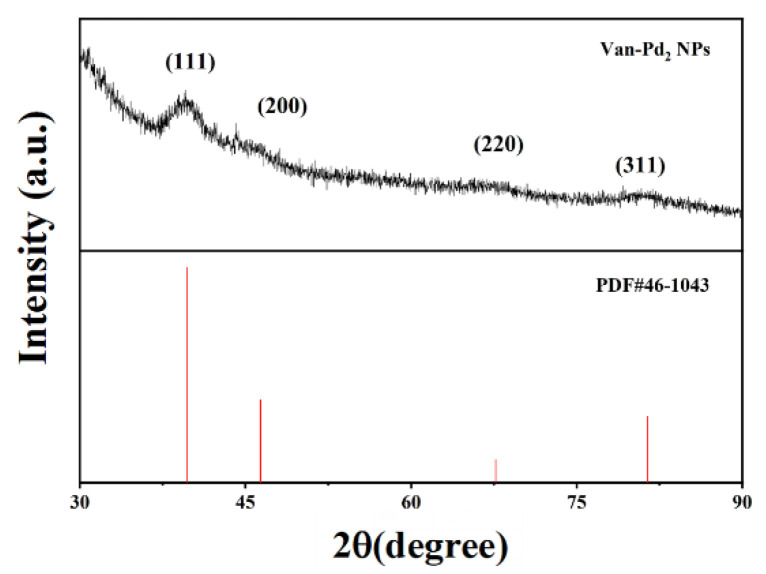
XRD diffraction pattern of Van-Pd_2_ NPs.

We selected Van-Pd_2_ NPs for subsequent XRD to analyze their composition and structure. The diffraction spectra of Figure 4 show that the 2θ values of Van-Pd_2_ NPs are 39.71°, 46.36°, 67.66°, and 81.42°, respectively. These diffraction angles corresponded to the (111), (200), (220), and (311) faces of Pd, respectively. Compared with the reference code 46-1043 of Pd, it can be seen that the diffraction angle is consistent with the diffraction angle in the reference code, so it can be proved that the nanozyme we synthesized contained the element Pd. Therefore, we have successfully synthesized Van-Pd_2_ NPs through the characterization of XRD.

### 3.2. Characterization of Peroxidase-like Activity

As a commonly used substrate for enzyme activity assays, TMB reacts rapidly to produce blue oxTMB in the presence of reactive oxygen species and a catalyst [28]. Then, UV-vis spectrophotometry was used to test the absorbance of the characteristic absorption peak of oxTMB and determine whether it retained a certain enzyme-like activity by comparing the absorbance.

**Figure 5:**
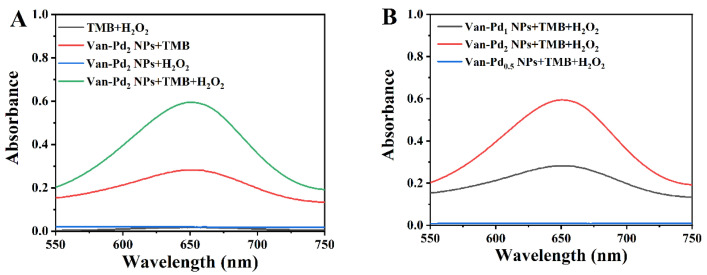
(**A**) Characterization of enzyme-like activity (reaction time: 5 min). (**B**) Comparison of peroxidase-like activity of Van-Pd_n_ NPs (n = 0.5, 1, 2).

To test whether Van-Pd_2_ NPs have oxidase-like activity and peroxidase-like activity, we designed the following control groups: TMB + H_2_O_2_, Van-Pd_2_ NPs + H_2_O_2_, TMB + Van-Pd_2_ NPs, and TMB + Van-Pd_2_ NPs + H_2_O_2_. It can be seen from Figure 5A that after 5 min of reaction, the TMB + Van-Pd_2_ NPs group and the TMB+ Van-Pd_2_ NPs + H_2_O_2_ group had a characteristic absorption peak of oxTMB at 652 nm. The TMB+ Van-Pd_2_ NPs + H_2_O_2_ group showed an excellent characteristic absorption peak at 652 nm; the absorbance was 0.60. The TMB + Van-Pd_2_ NPs group showed a characteristic absorption peak at 652 nm, but the absorbance was only 0.28. The characteristic absorption peak intensity at 652 nm of the TMB + Van-Pd_2_ NPs + H_2_O_2_ group was 2.14 times that of the only Van-Pd_2_ NPs group. This indicated that Van-Pd_2_ NPs had good peroxidase-like activity. In addition, TMB was oxidized to form oxTMB in the TMB + Van-Pd_2_ NPs group, indicating the oxidase-like activity of Van-Pd_2_ NPs.

From Figure 5B, it can be seen that when the amount of vancomycin species is the same, the greater the amount of Pd species, the better the peroxidase-like activity. Compared with the concentration of Pd species at 652 nm, the absorbance of Van-Pd_0.5_ NPs, Van-Pd_1_ NPs, and Van-Pd_2_ NPs reacted with TMB + H_2_O_2_ was 0.07, 1.04, and 1.10 after 5 min of reaction, respectively. It can be seen that the absorbance of the Van-Pd_2_ NPs + TMB + H_2_O_2_ group was the highest. This may be because at the same concentration of metal species, the less template used, the fewer active sites were covered, which was more conducive to the redox reaction. Therefore, Van-Pd_2_ NPs with the best peroxidase-like activity can be used for subsequent experiments.

**Figure 6:**
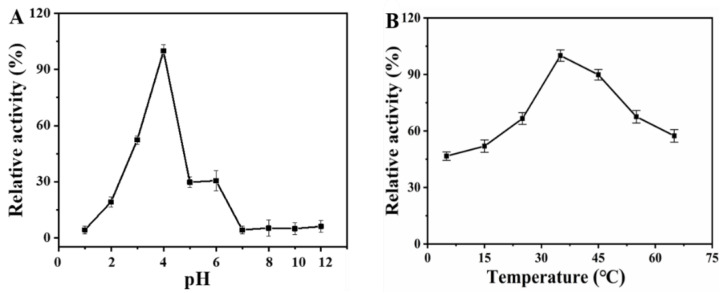
Characterization of peroxidase-like activity of Van-Pd_2_ NPs under different conditions: (**A**) pH; (**B**) temperature.

The peroxidase-like activity of Van-Pd_2_ NPs is affected by a number of external conditions, the main influencing factors of which are pH and temperature. Therefore, we need to investigate the peroxidase-like activity of nanozymes. As shown in Figure 6A, it can be seen that the peroxidase-like activity of Van-Pd_2_ NPs is set at 100% at pH = 4, and the peroxidase-like activity decreases significantly at other pHs. In addition, Figure 6B is an exploration of the optimal temperature for the peroxidase-like activity of Van-Pd_2_ NPs. Van-Pd_2_ NPs have the best peroxidase-like activity at 35 °C. Therefore, we can determine that the optimal conditions for Van-Pd_2_ NPs are pH 4 and a temperature of 35 °C.

### 3.3. Characterization of Van-Pd_2_ NPs Catalytic Kinetics

**Figure 7:**
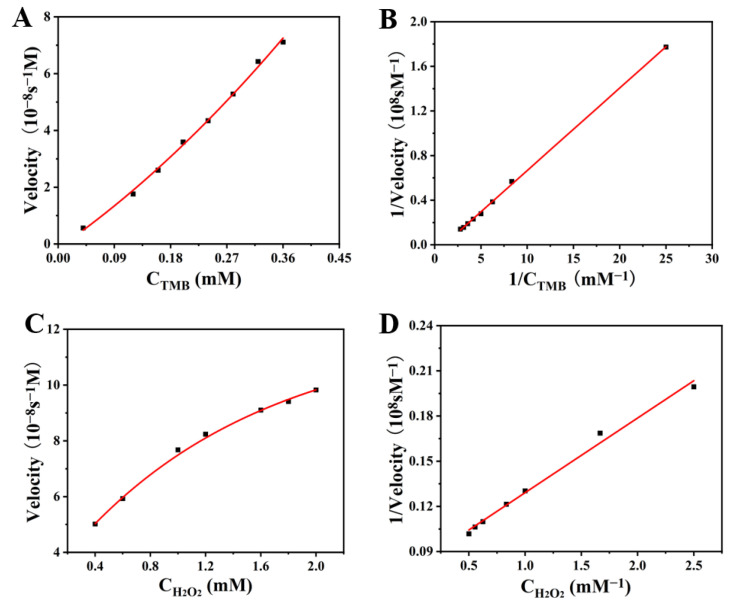
The catalytic kinetics of Van-Pd_2_ NPs for (**A**) TMB and (**C**) H_2_O_2_; (**B**,**D**) was the reciprocal of (**A**,**C**).

In order to explore the catalytic activity of peroxidase-like Van-Pd_2_ NPs, it is required to study their catalytic reaction kinetic characterization. The reaction kinetics of nanozymes are determined by changing the concentrations of the substrates TMB and H_2_O_2_. The test data were analyzed using the Lineweaver-Burk equation to obtain the data in Figure 7. Moreover, the peroxidase-like activity of other materials was compared with Van-Pd_2_ NPs shown in Table 1.

The Michael’s constant *K_m_* and the maximum reaction rate *V_max_* are then calculated according to the equation [34]. Firstly, the concentration of substrate TMB was changed within 0.04–0.4 mM, and the *K_m_* value and *V_max_* value of Van-Pd_2_ NPs were 1.007 mM and 13.6 × 10^−8^ Ms^−1^, respectively. Then, the concentration of H_2_O_2_ was changed within 0.2–2.0 mM, and the *K_m_* value and *V_max_* value of Van-Pd_2_ NPs were 0.623 mM and 12.566 × 10^−8^ Ms^−1^, respectively. Table 1 is a comparison of the catalytic performance of different nanozymes. Compared with other nanozymes, such as Au-NCs and Pb^2+^ for H_2_O_2_, the *K_m_* value of Van-Pd_2_ NPs for H_2_O_2_ is small. This indicates that Van-Pd_2_ NPs have good H_2_O_2_ affinity, which is conducive to the formation of reactive oxygen species and promotes the subsequent catalytic reaction. Thus, Van-Pd_2_ NPs have good peroxidase-like catalytic kinetics.

### 3.4. Mechanism of Van-Pd_2_ NPs Peroxidase-like Activity

**Figure 8:**
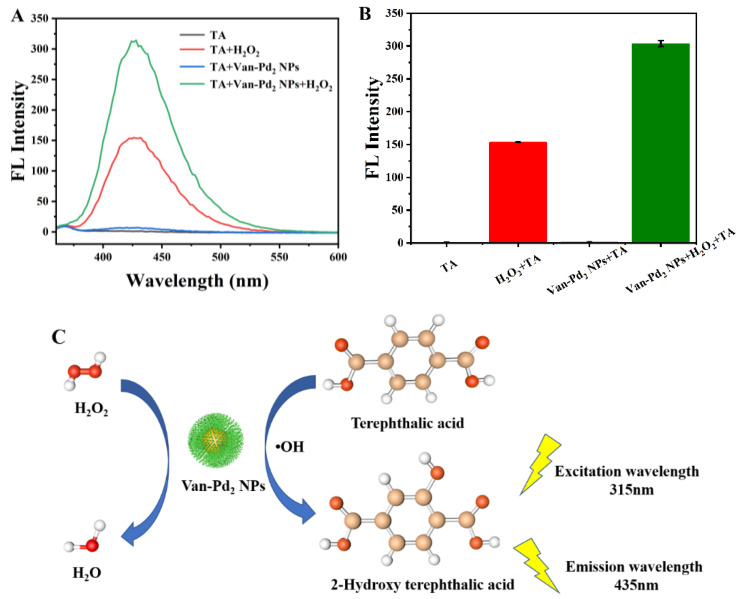
(**A**) Fluorescence intensity graphs of different experimental groups; (**B**) Histograms of fluorescence intensity at 435 nm; (**C**) TA detection •OH free radical diagram.

To delve into the mechanism of the catalytic process, additional research has been conducted. Figure 8C shows the fluorescence spectrum of the reaction mixture containing terephthalic acid (TA) and Van-Pd_2_ NPs after the addition of hydrogen peroxide (H_2_O_2_). Terephthalic acid is commonly used as a fluorescent probe to detect the presence of hydroxyl radicals (•OH). In the presence of •OH, terephthalic acid undergoes a reaction to form 2-hydroxy terephthalic acid (TAOH), which exhibits strong fluorescence at 435 nm when excited at a 315 nm wavelength. This fluorescence emission at 435 nm indicates the formation of TAOH due to the reaction between •OH and terephthalic acid.

The purpose of using terephthalic acid as a probe in this experiment is to investigate whether •OH radicals are produced by Van-Pd_2_ NPs through the catalytic decomposition of hydrogen peroxide. The fluorescence signal at 435 nm confirms the presence of •OH radicals, suggesting that the peroxidase-like activity of Van-Pd_2_ NPs is indeed due to the generation of •OH radicals. As depicted in Figure 8A, upon the addition of Van-Pd_2_ NPs into the solution containing TA and H_2_O_2_, it was observed that the fluorescence intensity curve of TAOH exhibited a notably higher value compared to the other groups. In Figure 8B, The peak value obtained from the group TA + H_2_O_2_ + Van-Pd_2_ NPs was measured at 304, which was approximately double the peak value of the TA + H_2_O_2_ group without Van-Pd_2_ NPs. These experimental findings strongly indicate that the catalytic mechanism of Van-Pd_2_ NPs primarily involves the generation of •OH.

### 3.5. HQ Detection

**Figure 9:**
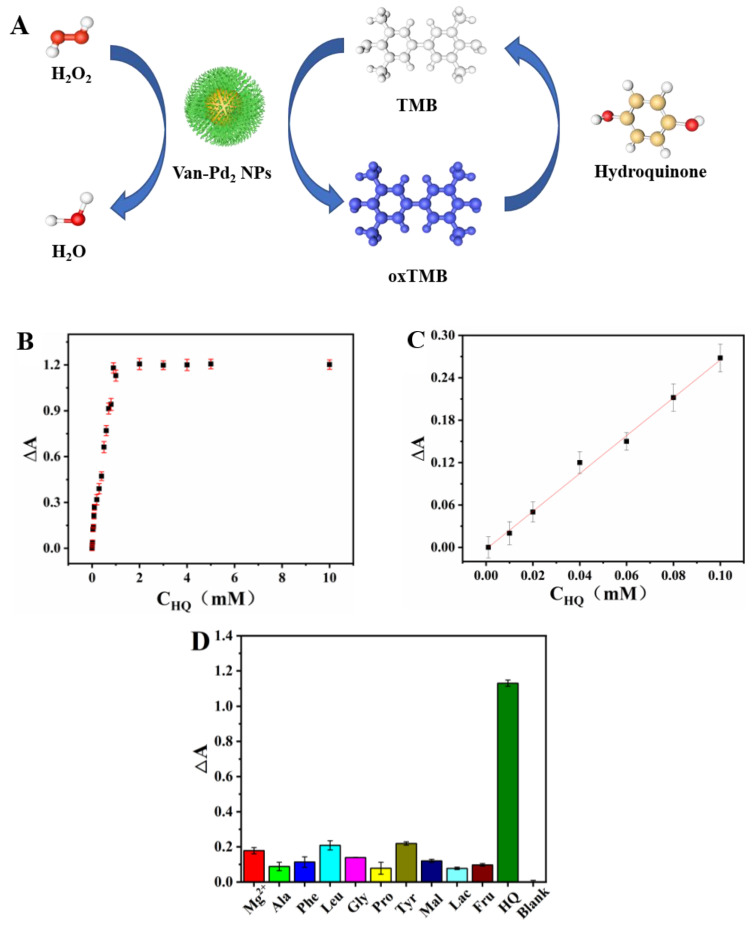
(**A**) Schematic diagram of Van-Pd_2_ NPs for colorimetric detection of HQ; HQ detection: (**B**) the plot of the absorbance difference (ΔA) of HQ; (**C**) linear fit plots of ΔA with different HQ concentrations; (**D**) selective detection of HQ.

HQ is a reducible biomass that reduces oxTMB to TMB. Therefore, we can use the excellent peroxidase-like activity of Van-Pd_2_ NPs to establish the standard curve for HQ detection. This experimental system is Van-Pd_2_ NPs + TMB + H_2_O_2_ + HQ, and then UV-vis spectrophotometry is used to detect the UV-vis absorption spectrum of the solution, as shown in Figure 9A.

**Table 2:** Comparison of HQ detection range and detection limits for different materials.

Materials	Detection Method	Linear Range (μM)	LOD (μM)	Reference
Van-Pd_2_ NPs	Colorimetry	1–100	0.323	this work
CuS–MoS_2_	Colorimetry	0.4–50	3.68	[35]
GCN-Cu NFs	Colorimetry	0.82–100	0.82	[36]
ZZFO/GF	Colorimetry	0–150	3.75	[37]
Au/CuO	Colorimetry	5–200	3	[38]
Pt/C-60/PGE	Electrochemical	50–1100	2.19	[39]
GCE/ErGO-cMWCNT/AuNPs	Electrochemical	1.2–170	0.39	[40]
Co_3_O_4_/MWCNTs	Electrochemical	10–800	5.6	[41]

The absorbance at 652 nm was measured by UV-vis spectrophotometry. As shown in Figure 9B, the absorbance at 652 nm gradually increases in a linear manner with the increasing concentration of HQ. However, when the concentration of HQ solution is greater than 1 mM, the absorbance at 652 nm tends to be stable. Figure 9C shows that the standard equation for HQ detection was Y = 0.0313 + 2.6812 × C_HQ_ (R^2^ = 0.9973). As shown in Table 2, the linear range was 0.001–0.1 mM, and the detection limits were 0.323 μM. Compared to the reported GCN-Cu NFs [36], the detection range is 0.82–100 μM, and the detection limit is 0.82 μM. Van-Pd_2_ NPs have high sensitivity and a wide detection range. Mg^2+^, alanine, phenylalanine, leucine, glycine, proline, glutamic acid, maltose, lactose, and fructose were used to test the selectivity of this assay. The other components caused very weak absorbance changes, as depicted in Figure 9D. These findings indicate that the method employed in the study exhibited high selectivity for the detection of glutathione.

In addition, Van-Pd_2_ NPs can be used to determine the recovery of samples. The sample is first added to different solutions to make a spiked solution. A certain amount of spiked solution was added to the reaction solution of Van-Pd_2_ NPs + TMB + H_2_O_2_. The absorbance of the solution was determined using UV-vis spectrophotometry. The spike recovery formula was then used to determine the recovery of the sample. From the comparison of the data in Table 3, it can be seen that the detection and recovery of HQ in seawater and river water are 108% and 98%, respectively. Therefore, the detection of HQ by Van-Pd_2_ NPs had high accuracy.

### 3.6. Biocompatibility

**Figure 10:**
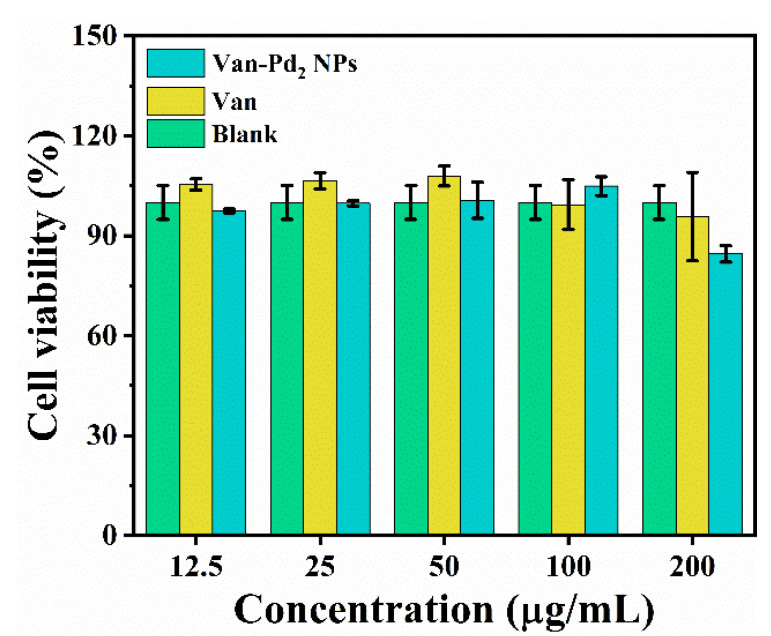
Cell viability of Van-Pd_2_ NPs.

Biological vancomycin was used in the preparation of Van-Pd_2_ NPs. In addition to testing the nanozyme activity and catalytic performance of Van-Pd_2_ NPs, it is also necessary to determine the biocompatibility of Van-Pd_2_ NPs. The MTT method is a commonly used method for determining the biocompatibility of samples. Van-Pd_2_ NPs with A549 cells were incubated for 24 h. Then, the cell viability of Van-Pd_2_ NPs was determined by MTT to determine the biocompatibility of nanozymes. The results are shown in Figure 10, and we set the Van-Pd_2_ NPs group, the vancomycin group, and the blank group. The cell viability in the vancomycin group remained at 90%, and the cell viability in the Van-Pd_2_ NPs group reached 85% at 200 μg/mL. By comparison with the cell activity of the blank group, Van-Pd_2_ NPs and vancomycin have almost no cytotoxicity. Therefore, it is known that Van-Pd_2_ NPs have good biocompatibility through cell viability assays.

## 4. Conclusions

In summary, we successfully synthesized Van-Pd_2_ NPs with good peroxidase-like activity by the biological template method. The catalytic kinetics of Van-Pd_2_ NPs conformed to the typical Michaelis–Menten equation, and there is a good affinity for H_2_O_2_ in peroxidase-like activity. The prepared nanozyme Van-Pd_2_ NPs were used to establish a simple and reliable detection method for HQ. The detection range was determined to be 1–100 μM, with a detection limit of 0.323 μM. Van-Pd_2_ NPs and vancomycin were non-cytotoxic. Therefore, the colorimetric detection method with high selectivity has a good application prospect in the detection of HQ.

## Figures and Tables

**Table 1:** Comparison of kinetic parameters *K_m_* and *V_max_*.

Materials	*K_m_* (mM)		*V_max_* (10^−8^ Ms^−1^)		Reference
	TMB	H_2_O_2_	TMB	H_2_O_2_	
Van-Pd_2_ NPs	1.007	0.623	13.611	12.566	this work
HRP	0.434	3.70	10.0	8.71	[29]
Au-NCs and Pb^2+^	0.58	30	4.13	3.39	[30]
Cu-Ag/rGO	0.63	8.62	4.25	7.01	[31]
CWNSs	0.053	4.25	17.09	20.06	[32]
NGZF	0.907	115.25	9.71	7.44	[33]

**Table 3:** Recovery of HQ detection in different samples.

Sample	Added HQ Concentration (μM)	Found HQ Concentration (μM)	Recovery (%)	RSD (%)
Running water	50	49	98	0.54
Seawater	50	54	108	1.25

## Data Availability

The data presented in this study are available upon request from the corresponding authors.
